# Fruit sugar hub: gene regulatory network associated with soluble solids content (SSC) in *Prunus persica*

**DOI:** 10.1186/s40659-024-00539-5

**Published:** 2024-09-06

**Authors:** Gerardo Núñez-Lillo, Victoria Lillo-Carmona, Alonso G. Pérez-Donoso, Romina Pedreschi, Reinaldo Campos-Vargas, Claudio Meneses

**Affiliations:** 1 Escuela de Agronomía, Facultad de Ciencias Agronómicas y de los Alimentos, Pontificia Universidad Católica de Valparaíso, Quillota, Chile; 2 Departamento de Fruticultura y Enología, Facultad de Agronomía y Sistemas Naturales, Pontificia Universidad Católica de Chile, Santiago, Chile; 3 Facultad de Ciencias Biológicas, Pontificia Universidad Católica de Chile, Santiago, Chile; 4 Millennium Institute Center for Genome Regulation (CRG), Santiago, Chile; 5 Departamento de Producción Agrícola, Facultad de Ciencias Agronómicas, Universidad de Chile, Santiago, Chile; 6 ANID-Millennium Science Initiative Program – Millennium Nucleus for the Development of Super Adaptable Plants (MN-SAP), Santiago, Chile

**Keywords:** Peach, Sweetness, Sugar accumulation, RNA-seq, Gene network, *PpSWEET15*, *PpC3H67*

## Abstract

Chilean peach growers have achieved worldwide recognition for their high-quality fruit products. Among the main factors influencing peach fruit quality, sweetness is pivotal for maintaining the market's competitiveness. Numerous studies have been conducted in different peach-segregating populations to unravel SSC regulation. However, different cultivars may also have distinct genetic conformation, and other factors, such as environmental conditions, can significantly impact SSC. Using a transcriptomic approach with a gene co-expression network analysis, we aimed to identify the regulatory mechanism that controls the sugar accumulation process in an 'O × N' peach population. This population was previously studied through genomic analysis, associating LG5 with the genetic control of the SSC trait. The results obtained in this study allowed us to identify 91 differentially expressed genes located on chromosome 5 of the peach genome as putative new regulators of sugar accumulation in peach, together with a regulatory network that involves genes directly associated with sugar transport (*PpSWEET15*), cellulose biosynthesis (*PpCSLG2*), flavonoid biosynthesis (*PpPAL1*), pectin modifications (*PpPG*, *PpPL* and *PpPMEi*), expansins (*PpEXPA1* and *PpEXPA8*) and several transcription factors (*PpC3H67*, *PpHB7*, *PpRVE1* and *PpCBF4*) involved with the SSC phenotype. These results contribute to a better understanding of the genetic control of the SSC trait for future breeding programs in peaches.

**Supplementary Information:**

The online version contains supplementary material available at 10.1186/s40659-024-00539-5.

## Introduction

The Chilean fresh fruit export industry has achieved worldwide recognition for its high-quality fruit products, particularly grapes, apples, tomatoes, cherries, and peaches. In peach production, 302,578 tons of Chilean fresh peaches and nectarines were reported to be produced in 2021 [6]. Sweetness is a pivotal attribute among the myriad factors influencing fruit quality. The quantification of sweetness in fruit is fundamentally grounded in the soluble solids content (SSC), which encompasses all soluble substances within the fruit, primarily sugars [20]. Achieving optimal sweetness levels, which entail a harmonious balance between sugars, acids, and other chemical components, has become a defining factor in maintaining the competitiveness of peaches and nectarines in the market.

However, fruit development is accompanied by other quality-related processes like growth, softening, or flesh color development [9]. Studies focused only on the SSC phenotype are a great challenge, considering that all these processes, including sugar accumulation (SSC), develop parallel until the fruit is harvested. In peach, it has been reported that there could be a pleiotropic effect between SSC and maturity date phenotypes [5]. It has also been observed that SSC is often linked to the glabrous trait (G) being nectarines sweeter than peaches [16], and can even be influenced by environmental factors or field practices such as temperature, radiation, photoperiod, precipitation, irrigation, fertilization, rootstock-scion interactions, pruning, and canopy management [3]. In related species such as apricots, an association between sugar accumulation and fruit flesh color development has been reported, identifying potential biomarkers in both phenotypes' carotenoids, starch, and sucrose metabolism pathways [8].

Despite these difficulties, numerous studies have been carried out to determine SSC genetic control in peaches using many peach varieties via QTL analysis approaches. Multiple genomic regions were identified for the SSC trait; for example, Eduardo et al. [5] associated the SSC trait with linkage group 4 (LG4) at Pp4:10,222,334 of the peach genome in two peach populations, 'B × O' ('Bolero' × 'OroA') and 'C × A' ('Contender' × 'Ambra'). On the other hand, Zeballos et al. [30] identified QTLs for SSC in LG4 (Pp4:7,090,720..10,280,095) and LG5 (Pp5:5,813,029..10,280,095) in a 'V × B' population ('Venus' × 'Big Top'), Hernández-Mora et al. [10] identified one consistent QTL in LG4 (Pp4:11,208,348..12,107,192) and two consistent QTLs in LG5 (Pp5:1,376,476..6,071,714 and Pp5:15,249,345..18,236,498) using a multiple peach progenies approach, and Shi et al. [25] identified consistent QTLs for SSC in LG1 (four QTLs along the chromosome 1 between Pp1:13,177,641..42,673,647), LG4 (Pp4:28,137,742..30,186,866) and LG5 (Pp5:846,656..1,094,221) in a peach population from a cross between the 'Shahong' and 'Hongfurong' varieties, demonstrating complex genetic control for this phenotype within peach genetic diversity. However, it has also been observed that a large part of the genetic control of this phenotype is consistently controlled by LG5 (Pp5:12,106,999..18,240,259), for example, in an 'O × N' population from a cross between the 'O'Henry' and NR-053 varieties [21], in a multifamily approach carried out by Rawandoozi et al. [22] and in the work mentioned above by Hernández-Mora et al. [10].

On the other hand, several transcription factors are associated with SSC regulation in fruits. For example, the transcription factors *MdAREB2* [19] and *MdWRKY32* [14] are involved in the regulation of sugar accumulation and starch-sugar metabolism during fruit development in apple fruit (*Malus domestica*). The transcription factor *VvWRKY22* has been shown to regulate sugar accumulation in grape plants (*Vitis vinifera*) by interacting with the VvSnRK1.1 or VvSnRK1.2 proteins, which are sucrose nonfermenting protein kinases related to sugar metabolism [11]. Research on the transcription factors associated with SSC in peaches is relatively limited compared to that in other fruit crop species. However, the transcription factors *PpMYB98* [21], *PpCBF6* [2], *PpNAC1* and *PpNAC5* [31] have been associated with the regulation of fruit soluble sugar accumulation in peach fruit.

It is important to note that further research is needed to understand the transcriptional regulation of SSC in peach fruitfully. Different cultivars may also have distinct transcriptional networks governing sugar accumulation. Other factors, such as hormonal regulation and environmental conditions, can significantly impact SSC in peaches. In this research, we aimed to (i) identify the differentially expressed genes involved in the modulation of the SSC trait via transcriptomic analysis between contrasting SSC siblings of the 'O × N' population, (ii) evaluate the regulatory mechanisms that control the sugar accumulation process via gene network analysis, and (iii) validate candidate genes for the SSC phenotype in contrasting peach varieties.

## Materials and methods

### Plant material

The experimental design of this research work is divided into two parts: identifying candidate genes for the SSC phenotype by transcriptomic analysis and validating these candidate genes by RT-qPCR. On the one hand, a segregating population of peaches was used for transcriptomic and co-expression network analysis. At the same time, the results were validated in a different genetic background using peach varieties.

For transcriptomic analysis at the harvest stage associated with differences in soluble solid content (SSC) phenotype, an F1 population with 194 individuals obtained from a cross between 'O'Henry' and NR-053 ('O × N') directed by the Chilean Peach Breeding Program (Universidad de Chile-Andes New Varieties Administration) was evaluated during the 2015–2017 seasons [21]. A summary of the 'O × N' phenotyping information is shown in Additional file 1: Table S1. The 'O'Henry' variety produces melting yellow-flesh peach fruit with a low SSC, while NR-053 (Maillarmagie cv. Magique®) is a nectarine variety characterized by the production of high SSC melting fruit with white flesh. The 'O × N' population consists of eight-year-old trees grown on 'G × N' rootstock in an experimental orchard located in INIA-Rayentué (VI Region, Chile) and was previously used to construct a high-density genetic map and QTL analysis for fruit quality traits such as harvest date, SSC and mealiness [21].

The harvest stage was determined according to the index of absorbance difference (I_AD_), a nondestructively indirect determination of the chlorophyll content in the fruit skin, considering a good I_AD_ value to harvest between 0.8 and 1.2, as described by Lurie et al. [18]. Fruit quality traits such as fruit size, weight, color, and SSC were evaluated at the harvest stage, considering the average of five fruits per tree. SSC trait in ºBrix was recorded using a temperature-compensated refractometer (Atago, Tokyo, Japan). Six individuals of the 'O × N' population were selected and classified into two phenotypic classes according to their SSC phenotype at the fruit harvest stage during three evaluation seasons. In this sense, as biological replicates, three individuals were used as LowSSC samples (O×N-002, O×N-060, O×N-084), and other three individuals were used as HighSSC samples (O×N-037, O×N-184, O×N-194).

To evaluate the expression levels of the 'O × N' candidate genes for the SSC phenotype, three peach varieties ('Summer Fire,' 'Venus,' and 'Rebus') with contrasting SSC phenotypes were used for RT‒qPCR validation at the harvest stage. All these cultivars produce yellow-flesh nectarines and consist of 7-year-old trees grown on 'Nemaguard' rootstocks from the University of Chile Peach Improvement Program (Rinconada, Metropolitan Region, Chile). The harvest stage of each cultivar was determined considering an adequate firmness to harvest between 13–15 N, and the I_AD_ values were measured from already harvested fruit. Each variety was evaluated for fruit quality traits such as size, weight, color, and SSC, considering the average of five fruits during the 2020–2021 season.

### RNA extraction

For both transcriptomic and RT-qPCR analysis, total RNA was extracted from 100 mg of frozen fruit mesocarp at the harvest stage using a Spectrum™ Plant Total RNA kit (Sigma‒Aldrich, St. Louis, MO, USA) following the manufacturer's instructions and stored at − 80 °C. RNA quantity was evaluated with a Qubit® 2.0 fluorometer (Invitrogen™, Carlsbad, CA, USA) using a Qubit™ RNA BR assay kit. RNA integrity was assessed by capillary electrophoresis using an Automated CE Fragment Analyzer™ system (Agilent Technologies, Santa Clara, CA, USA) with the RNA kit DNF-471-0500 (15 nt). The RNA quality number (RQN) was used to determine RNA integrity for sequencing analysis. RNA samples with an RQN value greater than 7.0 were used for the following steps.

### RNA library construction and sequencing

For transcriptomic analysis, three selected 'O × N' individuals for each SSC phenotypic class (LowSSC and HighSSC) in triplicate at the harvest stage were used for library construction. RNA libraries were prepared with a TruSeq Stranded Total RNA Kit (Illumina, San Diego, CA, USA) following the manufacturer's instructions. The library concentration was determined with a Qubit®2.0 fluorometer (Invitrogen™) using a Qubit™ dsDNA BR assay kit, and the library size and integrity were evaluated by capillary electrophoresis using the Automated CE Fragment Analyzer™ system (Agilent Technologies) with the DNF-474-0500 HS NGS Fragment Kit. The constructed libraries were sequenced using Macrogen sequencing services (Seoul, Korea) in paired-end mode on a HiSeq4000 sequencer with a read length of 150 bp.

### Differential expression analysis

The raw sequencing data were evaluated using FASTQC software and filtered with trim-galore v0.6.7 software by applying the following criteria: (i) adapter sequences were removed; (ii) reads with a quality score < 25.0 were eliminated, and (iii) reads with a length < 50 nucleotides were eliminated. STAR aligner v2.7.10 software [4] was used to align the filtered reads against the *Prunus persica* v2.1 reference genome [28]. For each library, the *featureCounts* function from the Bioconductor-Rsubread package v2.8.1 [15] was applied to assign expression values to each uniquely aligned fragment. Differential gene expression analysis was performed using the Bioconductor-DESeq2 v1.34.0 package, and data normalization was performed according to the DESeq2 median of ratios method [17]. Differentially expressed genes (DEGs) with an FDR < 0.05 and a |log_2_FC| > 1.0 were selected for reliable network construction to explain the SSC phenotype. Principal component analysis of the transcriptomic data of all the normalized gene counts was performed using the ggfortify R package v0.4.14 [26] with the *autoplot* function, and a heatmap was constructed to visualize the DEGs via the pheatmap v1.0.12 package. To search for genetic processes and pathways overrepresented in the DEG lists, a genetic enrichment analysis was performed using the Genetic Ontology (GO) database with the R package ClusterProfiler v4.0.5 [29] using the *compareCluster* function.

### Soluble solids content network analysis

Network analysis was performed using the ConnecTF platform [1], an online database with transcription factor-target gene interaction information from Arabidopsis, maize, and rice that allows the identify putative regulatory gene co-expression networks available at https://connectf.org. The list of DEG candidates for SSC was used as a "Target Gene List" and "Filter TFs" to identify candidate transcription factors in the ConnecTF database for network construction. The Target List Enrichment tool was used to determine the significance of each transcription factor by comparing the target gene list and queried analyses considering a *p* < 0.01*.* In summary, the selection of candidate transcription factors was carried out (i) considering the number of target genes of each transcription factor, (ii) with a filter of *p* < 0.01 in the ConnecTF database, and (iii) with the most marked differences between low- and high-SSC individuals with a |log_2_FC| > 1.0. Finally, network construction was performed using Cytoscape software v3.9.1 [24].

### RT‒qPCR candidate gene evaluation in peach varieties

RT‒qPCR was used to analyze the transcript levels of five selected DEGs in three contrasting varieties for the SSC phenotype ('Summer Fire,' 'Venus' and 'Rebus'). For cDNA synthesis, 1 μg of total RNA was first treated with DNase I (Thermo Fischer Scientific, Waltham, MA, USA), and the Superscript II RT system (Invitrogen™) was used according to the manufacturer's instructions. cDNA synthesis was confirmed by 2.0% agarose gel electrophoresis. RT‒qPCR amplification reactions were performed in a total volume of 10.0 μL. The reaction mixture contained 1.0 μL of template cDNA, 0.5 μL of primers (0.25 μL of forward and 0.25 μL of reverse), 0.2 μL of ROX, 3.3 μL of nuclease-free water and 5.0 μL of SYBR Green PCR intercalating dye (Sigma‒Aldrich) as a fluorescent indicator. Reactions were performed using three biological and two technical replicates for each variety at the harvest stage in the AriaMx Real-Time PCR System (Agilent Technologies). Relative expression was calculated using the housekeeping gene *PpeRPII* as a reference gene [27]. The data were plotted and analyzed in GraphPad Prism v9.4.1. The correlation between the soluble solid content phenotype and the RT‒qPCR expression value of each transcript was determined via Pearson correlation analysis.

## Results

### Selection of contrasting 'O × N' siblings for soluble solids content and sequencing summary metrics

Six peach individuals from the 'O × N' population were selected for transcriptomic analyses of the soluble solids content (SSC) phenotype. These individuals were classified into two phenotypic classes (LowSSC and HighSSC), which presented consistent SSC values during the three evaluation seasons (2015–2017). Detailed phenotyping information about selected individuals in all evaluation seasons is shown in Additional file 2: Table S2, considering fruit pubescence (peach/nectarine), flesh color (white/yellow), maturity date, fruit size, firmness, and SSC. There were no significant differences in maturity date, fruit size, and firmness values between phenotypic classes, and no correlation between SSC traits was observed with flesh color or maturity date phenotypes. Significant differences were observed for SSC traits between phenotypic classes with no significant differences between biological replicates and evaluated seasons (Additional file 2: Table S2), with average SSC values of 10.4 ºBrix (LowSSC) and 17.0°Brix (HighSSC).

Interestingly, there is a correlation between SSC and the glabrous traits in selected 'O × N' individuals, with peaches associated with lower soluble solids content than nectarines (Additional file 2: Table S2). We constructed three RNA libraries for each individual chosen using different fruits from three independent RNA extractions to be sequenced as technical replicates. The sequencing results are detailed in Additional file 3: Table S3. On average, 40,331,623 reads were sequenced for each RNA library, and approximately 91.3% of the total reads passed quality filters. After filtering, 89.7% of the total sequenced reads were correctly aligned on average against the *Prunus persica* v2.1 reference genome.

### Differential expression analysis between 'O × N' siblings with low and high SSCs

**Fig. 1:**
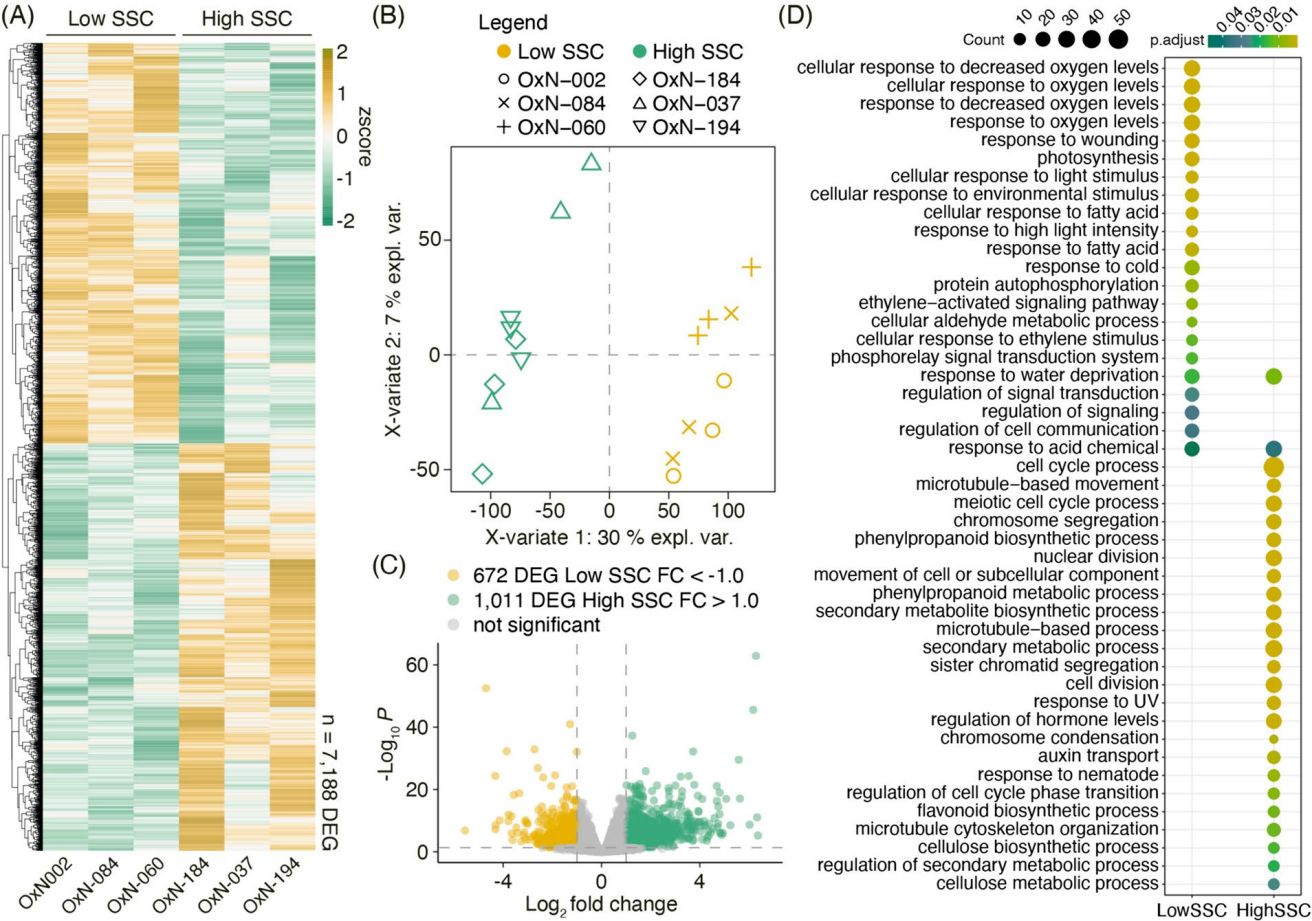
Differential expression analysis between contrasting SSC samples from the 'O × N' population. **A** Differentially expressed genes between LowSSC and HighSSC samples are represented in a green-yellow color scale heatmap (FDR < 0.05). Data was scaled using a z-score scaling method, dividing the mean value of each gene by the standard deviation. Each column represents the average expression of three replicates for each selected individual. **B** Partial least square discriminant analysis (PLS-DA) using normalized counts of each sequenced library. Each symbol represents the three replicates of each selected individual. Yellow and green symbols represent LowSSC and HighSSC samples. **C** Volcano plot with differentially expressed genes comparing LowSSC and HighSSC samples. Candidate genes for each group were selected with a *p-value* < 0.01 and |log_2_FC| > 1.0. **D** Gene ontology term enrichment analysis with genes overexpressed in LowSSC and HighSSC samples. The scale color represents the adjusted p-value, and the point size represents the gene ratio

When performing differential expression analysis between LowSSC and HighSSC samples, we identified 7188 differentially expressed genes (DEGs) with an FDR < 0.05, which are represented in a green–yellow color scale heatmap (Fig. 1A) considering the average expression value of the three technical replicates for each individual. The biological and technical replicate distributions are shown in Fig. 1B. The individual with the greatest dispersion among the technical replicates was O×N-037. However, even so, it was possible to differentiate both phenotypic classes, with the first component explaining 30% of the observed variability. To perform an SSC network analysis with the genes that presented more significant expression differences between phenotypic classes, the 7188 DEGs identified were filtered with a |log_2_FC| > 1.0, as shown in the volcano plot in Fig. 1C. Thus, 672 and 1011 candidate genes with higher expression in individuals with LowSSC and HighSSC were selected for the following analyses.

Gene Ontology analysis was carried out to identify enriched terms between both sets of candidate genes. As shown in Fig. 1D, the enriched GO terms in the group of 672 candidate genes for LowSSC related to photosynthesis, response to light stimulus, environmental stimulus, decreased oxygen levels, wounding, fatty acids, cold, ethylene, and water deprivation were identified. GO terms related to the cell cycle, cell division, chromosome segregation, phenylpropanoid, and flavonoid biosynthetic processes, regulation of hormone levels, auxin transport, and cellulose biosynthetic and metabolic processes were identified in the group of 1011 candidate genes for the HighSSC samples.

### Candidate regulatory gene identification comparing DEG physical positions with the localization of the described QTL for SSC in the 'O × N' population

According to the results published by Nuñez-Lillo et al. [21], the position of the QTL for the SSC phenotype in the 'O × N' population was found to be located between 12.1 and 18.3 Mbp of chromosome 5 of the peach genome containing a total of 1211 genes. Considering the physical position of the 1683 DEGs obtained in this research, only 91 colocalized with the QTL for SSC, 57 with greater expression values in HighSSC samples, and 34 with greater expression values in LowSSC samples. These 91 genes could be considered candidate regulators of this phenotype (Additional file 4: Table S4). Of this list of candidate genes, two cellulose metabolism-related genes, one member of the MAP kinase family of proteins, and nine transcription factor genes stand out as regulatory genes of the SSC phenotype due to their functional annotations, as shown in Table 1.

**Table 1:** Candidate regulatory genes are differentially expressed between contrasting SSC individuals colocated with the QTL for SSC in the 'O × N' population

GeneID	Description	AthalianaID	Normalized expression		
			LowSSC	HighSSC	log_2_(FC)
Prupe.5G123800	Cellulose synthase-like protein G1-related	AT4G23990	24.7	93.0	1.9
Prupe.5G131300	endo-1,4-β-glucanase	AT1G02800	4.5	29.4	2.7
Prupe.5G146100	No apical meristem (NAM) protein	AT5G13180	0.4	1.8	2.3
Prupe.5G158600	Zinc finger CCCH domain-containing protein 43	AT5G63260	1.8	5.0	1.5
Prupe.5G158700	Zinc finger CCCH domain-containing protein 43	AT5G63260	9.9	23.6	1.3
Prupe.5G191200	TCP family transcription factor	AT3G47620	0.8	2.6	1.6
Prupe.5G201400	C2H2-type zinc finger (zf-C2H2_6)	AT1G26610	1.3	4.8	1.9
Prupe.5G211200	Basic leucine-zipper 70-related	AT5G60830	6.5	18.3	1.5
Prupe.5G241000	Protein Reveille 1-related	AT1G18330	0.9	2.9	1.7
Prupe.5G130300	MYC	AT4G17880	5.2	0.9	-2.6
Prupe.5G149000	Squamosa promoter-binding protein 13A-related	AT5G50670	5.5	1.3	-2.1
Prupe.5G236900	Mitogen-activated protein kinase kinase 7-related	AT1G73500	65.7	31.0	-1.1

Among these 12 candidate genes, nine had relatively high expression in the HighSSC samples, and only three had relatively high expression in the LowSSC samples. The interaction information for the four transcription factors shown in Table 1 was obtained from the ConnecTF database (AT5G13180, AT5G63260, AT1G18330, and AT5G50670). The only gene with enough target genes represented in the differential expression analysis (*p*-value < 0.01) to be used in the SSC gene regulatory network construction for the 'O × N' population was the transcription factor AT5G63260, which is described in *Arabidopsis thaliana* as a C3H67. In the peach genome, this transcription factor was annotated as a zinc finger CH domain-containing protein 43 for two genes, Prupe.5G158600 and Prupe.5G158700, with higher expression values in HighSSC samples.

### Regulatory network analysis for the soluble solids content phenotype

In the group of candidate genes for LowSSC, a total of 14 transcription factors were identified in the transcription factor-target gene (TF-TG) interaction database, of which 8 had a *p* value > 0.01 based on the ratio of total target genes and differentially expressed genes (query); therefore, they were excluded from the network analysis. In the group of candidate genes for HighSSC, 16 transcription factors were identified, 13 of which were excluded from the network analysis due to a *p* value > 0.01. In this sense, six transcription factors associated with individuals with LowSSC (*PpCBF4*, *PpHB40*, *PpSTZ*, *PpESE3*, *PpERF4*, and *PpERF017*) and three transcription factors related to individuals with HighSSC (*PpRVE1*, *PpHB7*, and *PpC3H67*) were selected for SSC network construction.

**Fig. 2:**
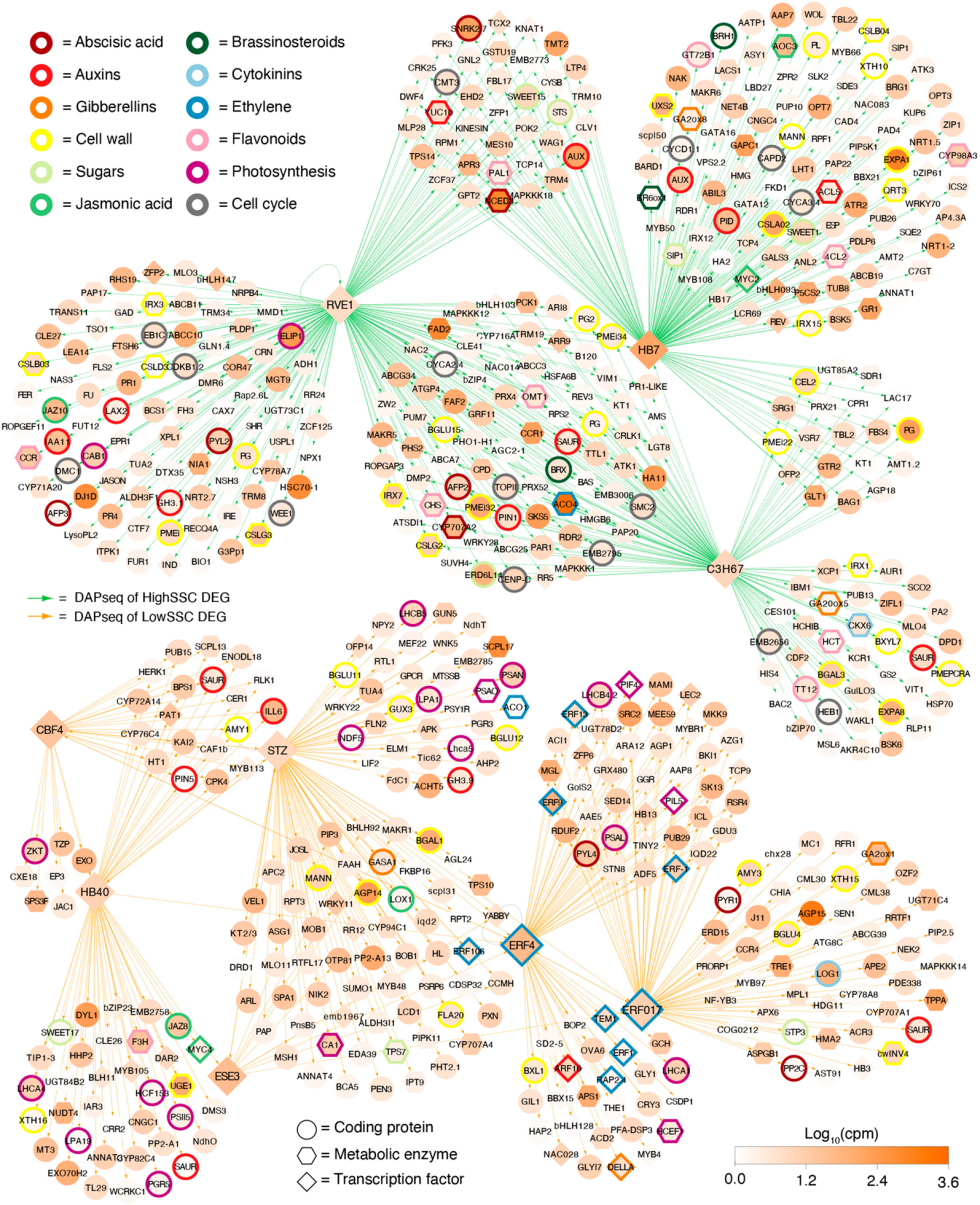
Soluble solids content network analysis. Representation of most informative genes associated with soluble solids content regulatory network. Each node represents a differentially expressed gene, and each edge represents a DAP-seq gene association. Orange-scaled colored nodes correspond to the fold change absolute value between Low SSC and High SSC comparison. Nodes with colored borders correspond to genes associated with metabolic pathways or signaling

A regulatory network for the SSC phenotype was constructed with these nine transcription factors and the other 620 DEGs with TF-TG interactions in the ConnecTF database (Fig. 2). The HighSSC samples showed a high number of transcripts with functions related to the cell cycle (*PpCYCA2.4*, *PpCYCA3.4*, *PpCYCD1.1,* and *PpCDKB1.2*), flavonoid biosynthesis (*PpPAL1*, *PpCHS*, *Pp4CL2*, *PpHCT,* and *PpCCR*), and the regulation of brassinosteroids (*PpBRX*, *PpBRH1,* and *PpBR6ox1*). On the other hand, with higher expression in LowSSC samples, a high number of transcripts with functions related to photosynthesis (*PpPSAO*, *PpPSAN*, *PpPSAL*, *PpPIL5*, *PpPIF4*, *PpNDF5* and several *PpLHC*s) and ethylene pathways (*PpACO1*, *PpERF1*, *PpERF4*, *PpERF9*, *PpERF13*, *PpERF17*, *PpERF106* and *PpRAP2.4*) were identified.

Furthermore, several metabolic pathways, including sugar accumulation, cell wall remodeling, and regulation of abscisic acid, jasmonic acid, and auxin pathways, were represented in both the LowSSC and HighSSC samples. However, regarding genes related to sugar accumulation, a more significant number of genes were detected in HighSSC samples (*PpSWEET1*, *PpSWEET15*, *PpSTS*, *PpERD6L14, and PpSIP1)* than in LowSSC samples *(PpSWEET17, PpTPS7,* and *PpSTP3*). Finally, the cell wall remodeling-related genes identified in the HighSSC and LowSSC samples differed markedly. On the one hand, in HighSSC individuals, a greater number of genes associated with cellulose biosynthesis (*PpIRX1*, *PpIRX3*, *PpCSLA02*, *PpCSLB03*, *PpCSLB04*, *PpCSLD3*, *PpCSLG2,* and *PpCSLG3*) and pectin modifications (*PpPL*, *PpQRT3*, several *PpPG*s and several *PMEi*s) were identified along with two genes described as expansins (*PpEXPA1* and *PpEXPA8*). On the other hand, three genes with β-glucosidase activity (PpBGLU4, PpBGLU11, and *PpBGLU12*) and two xyloglucan endotransglucosylases/hydrolases activity (*PpXTH15* and *PpXTH16*) associated with the hemicellulose disassembly process were identified in LowSSC individuals.

### RT‒qPCR validation of five candidate genes in SSC-contrasting peach varieties

**Fig. 3:**
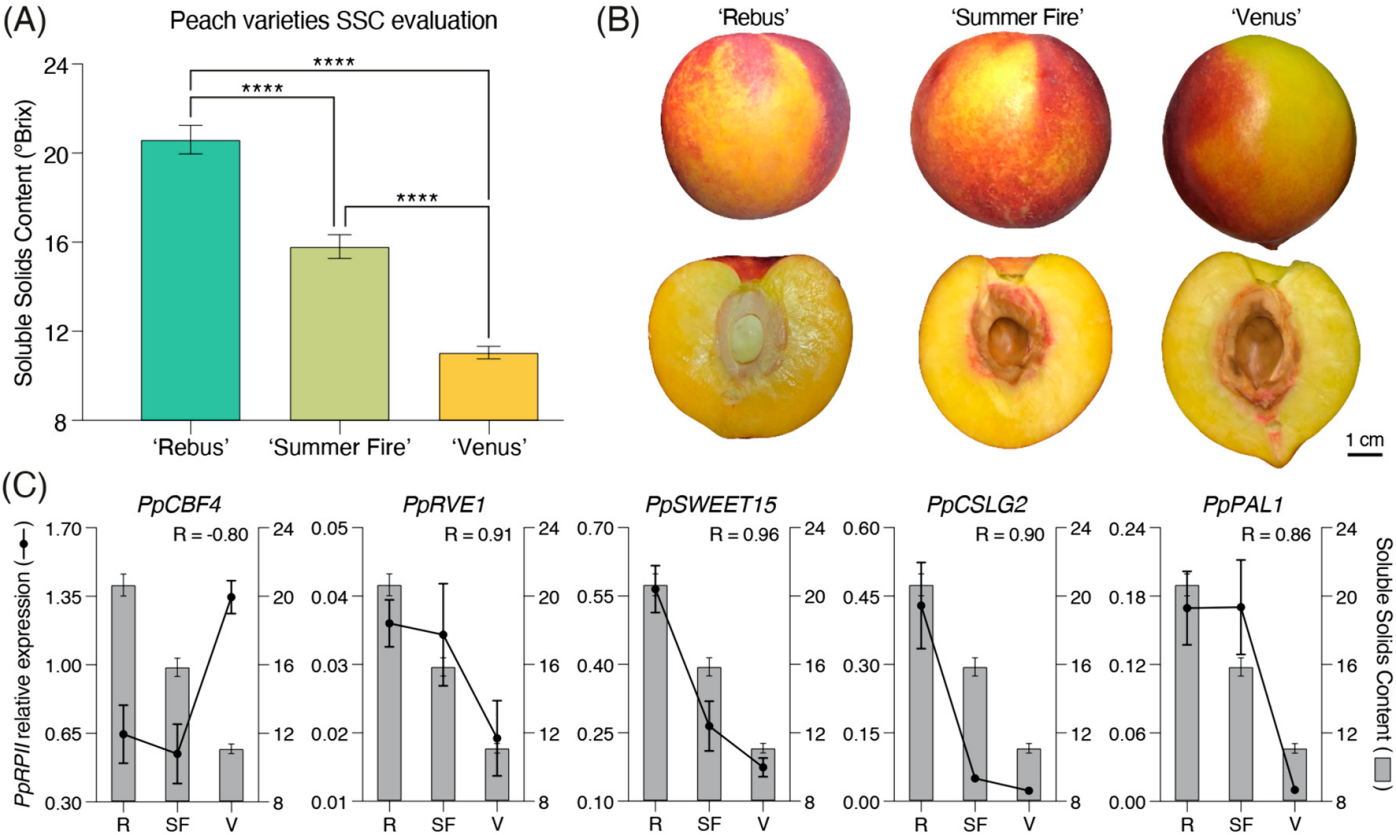
Candidate gene validations by RT-qPCR in contrasting peach varieties for the SSC trait. **A** Characterization of SSC phenotype in three peach varieties, 'Rebus' (R), 'Summer Fire' (SF), and 'Venus' (V). Statistical analysis was performed with a one-way ANOVA test; significant differences were represented by an asterisk (****; *p* < 0.0001). **B** Fruit phenotype of the contrasting peach varieties for the SSC trait. Photographic record of the fruits' external (upper images) and internal (lower images) phenotypes in each selected variety. **C** Correlation analysis between each selected candidate gene's RT-qPCR expression values (left axis) and contrasting peach varieties' SSC phenotype (right axis)

Three contrasting peach varieties for the SSC trait were selected to evaluate and validate candidate gene associations with SSC genetic control ('Rebus,' 'Summer Fire' and 'Venus'). As shown in Fig. 3A, the three selected varieties had significant differences in soluble solids content, with the highest being 'Rebus', with an average SSC value of 20.7°Brix, followed by 'Summer Fire', with 15.9°Brix, and finally 'Venus', with the lowest SSC value (11.1°Brix). As shown in Fig. 3B, all these peach varieties exhibited similar phenotypes for other fruit quality traits, such as fruit size, skin color, pubescence, and flesh color. Therefore, these three peach varieties were suitable for SSC candidate gene validation, considering that HighSSC and LowSSC individuals in the 'O × N' population have average SSC values of 17.0 and 10.4°Brix, respectively.

To validate the results obtained in the 'O × N' population, five candidate genes from the SSC network analysis shown in Fig. 2 were analyzed by RT‒qPCR in the three contrasting SSC peach varieties described above, four with high expression in the HighSSC samples (*PpRVE1*, *PpSWEET15*, *PpCSLG2* and *PpPAL1*) and one with high expression in the LowSSC samples (*PpCBF4*). As shown in Fig. 3C, candidate genes with high expression in HighSSC samples of the 'O × N' population also presented high expression in the variety with the highest soluble solids content, 'Rebus.' By comparing the expression values obtained by RT‒qPCR of each selected transcript with the °Brix of each variety SSC phenotype, Pearson correlations of 0.91 (*PpRVE1*), 0.96 (*PpSWEET15*), 0.90 (*PpCSLG2*) and 0.86 (*PpPAL1*) were obtained. Similarly, the expression of *PpCBF4,* the only evaluated candidate gene with high expression in LowSSC samples, was greater in the 'Venus' variety according to RT‒qPCR, with a Pearson correlation of -0.80 with the SSC phenotype. These results validated the relationships of all these candidate genes with the observed differences in the SSC phenotype.

## Discussion

The 'O × N' peach population used in this research for transcriptomic analysis was evaluated for 3 years for different fruit quality traits, including soluble solids content [21]. During the 2015–2017 seasons, the 'O × N' population was segregated for the SSC trait, with values between 8 and 25°Brix. It has been reported that fruit sugar accumulation is highly affected by other fruit developmental phenotypes such as pubescence, flesh color development, or maturity date. The selection of 'O × N' individuals was carried out considering that the phenotypic classes (LowSSC and HighSSC) did not have significant differences for these traits (Additional file 2: Table S2). However, in the 'O × N' population, it was impossible to identify a substantial number of nectarines individuals with low SSC or peaches with high SSC. This relationship between fruit pubescence and soluble solids content has been previously described in peaches [23]. This is probably because the genomic regions that control both traits are very close in the peach genome.

In previous research, the SSC phenotype in the 'O × N' population was associated with linkage group 5 of the peach genome through QTL analysis (Pp05:12,106,999–18,240,259). Interestingly, the results obtained by [21] show that the identified QTL for SSC colocalizes with the morphological marker for the glabrous trait (peach/nectarine), which explains why it was not possible to separate these phenotypes in the selection of individuals for transcriptomic analysis. Additionally, the parents 'O'Henry' and NR-053 genomes were previously sequenced. A variant detection analysis identified five candidate genes (colocated with the QTL for SSC) with nonsynonymous DNA variations that could explain the differences in sugar accumulation observed in the 'O × N' population. One was described as a probable polygalacturonase (*PpPG*), one as a sucrose synthase 6 (*PpSUS6*), and three annotated as transcription factors (*PpbHLH14*, *PpMYB98*, and *PpMADS6*).

According to the differential expression analysis between samples with contrasting SSC phenotypes performed in this research, we postulate that the 12 DEGs shown in Table 1 that colocalize with the QTL for SSC in the 'O × N' population could be added to the group of possible regulatory genes of the SSC phenotype along with the transcription factors *PpbHLH14*, *PpMYB98* and *PpMADS6* described in Nuñez-Lillo et al. [21]. However, more analyses are necessary to corroborate the participation of any of these candidate genes in the regulation of sugar accumulation in peach trees.

On the other hand, the three transcription factors with information in the ConnecTF database highly expressed in HighSSC individuals used to construct the SSC regulatory network shown in Fig. 2 are located on chromosomes Pp02 (*PpHB7*), Pp03 (*PpRVE1*) and Pp05 (*PpC3H67*) of the peach genome. The only gene colocalizing with the QTL for SSC in the 'O × N' population was the transcription factor *PpC3H67*, located at Pp05:14,060,293.14,065,599. Therefore, it is one of the most robust candidates for regulating the SSC trait in peach, although no functional information about this transcription factor has been published.

Figure 2 shows higher expression values of sugar accumulation-related genes (*PpSWEET1* and *PpSWEET15*) identified in samples with high SSC phenotypes. These genes are bidirectional membrane transporters that facilitate sugar transport along a concentration gradient and are involved in different cellular processes, such as pollen nutrition, nectar secretion, seed-filling plant-pathogen interactions, abiotic stress responses, and fruit development [12]. In particular, it has been reported that *MdSWEET15,* together with *MdSWEET9,* participates in the sugar accumulation process in apples during fruit development [32], while in tomatoes, it has been reported that *SlSWEET1*, -*2*, -*7,* and -*14* are involved in the early stage of the sugar accumulation process and that their expression decreases with maturation [7]. Ko et al. [13] confirmed that SlSWEET15 mediates apoplastic sucrose unloading from the phloem for carbon supply during fruit expansion and development, associating it with the control of sucrose accumulation in the fruit. Although one SWEET gene (*PpSWEET17*) was also detected in LowSSC individuals (Fig. 2), the expression levels of this gene are considerably lower than those of the SWEET genes identified in HighSSC individuals (approximately 10 times greater for *PpSWEET1* and *PpSWEET15* than for *PpSWEET17*). Furthermore, as shown in Fig. 3, the correlation of the *PpSWEET15* gene expression values in peach varieties with contrasting SSC phenotypes was evaluated (showing a correlation of 0.96), validating its relationship with peach sugar accumulation. Finally, for these reasons, we believe that *PpSWEET15* and *PpSWEET1* could be responsible for the observed differences in the SSC trait in peach trees.

On the other hand, many genes related to the flavonoid biosynthesis pathway and the cell wall remodeling process were also associated with HighSSC individuals, suggesting a correlation between these processes and the SSC trait in the 'O × N' population. These correlations were evaluated in contrasting SSC peach varieties using selected candidate genes (*PpPAL1* for flavonoid accumulation and *PpCSLG2* for cell wall remodeling). Both candidates showed strong correlations with the SSC trait in peach varieties (Fig. 3), demonstrating that there is a correlation between the SSC phenotype and the biosynthesis of flavonoids and cellulose in peach fruit varieties and not only in the 'O × N' segregating population. Similar results were reported by García-Gómez et al. [8] in apricot (*Prunus armeniaca* L.), flavonoid biosynthesis-related genes were identified differentially expressed between genotypes with contrasting SSC phenotype, among which are mentioned the *PAL*, *C4H*, *4CL,* and *DFR*. However, the regulatory mechanisms that control these correlations are still unknown, and more in-depth analyses are needed to corroborate these findings.

## Conclusion

The study utilizing the ‘O × N’ peach population to identify the regulatory mechanisms of soluble solids content (SSC) builds upon previous genomic studies that identified a consistent QTL for SSC on chromosome 5 of the peach genome. Our transcriptomic analysis of SSC traits in ‘O × N’ contrasting individuals led to identifying 91 DEGs as potential SSC regulatory candidate genes, colocalizing with the aforementioned QTLs. Additionally, a gene co-expression network analysis using the ConnecTF transcription factor-target gene interaction (TF-TG) database identified 629 DEGs as candidates associated with SSC regulation.

In HighSSC individuals, there was an overrepresentation of genes associated with flavonoid biosynthesis, sugar accumulation, cellulose biosynthesis, and pectin modification. Conversely, LowSSC individuals showed a greater representation of genes related to photosynthesis, the ethylene pathway, and hemicellulose disassembly processes. These findings suggest distinct molecular pathways contributing to the SSC phenotype in peaches. The implications of these results for peach production and breeding are significant. By identifying and validating several candidate genes for the SSC phenotype in contrasting peach varieties through RT-qPCR, such as PpSWEET15, PpCSLG2, PpPAL1, PpRVE1, and PpCBF4, we provide valuable genetic targets for future breeding programs aimed at improving fruit quality. These genes can be utilized as molecular markers to select for peach varieties with enhanced sweetness, thereby increasing the competitiveness of Chilean peach growers in the global market. Moreover, understanding the regulatory networks controlling SSC can aid in developing strategies to manipulate these pathways, producing consistently high-quality peaches under varying environmental conditions.

In summary, this study contributes to a deeper understanding of the genetic control of SSC in peaches, offering practical applications for breeding programs focused on fruit quality improvement.

## Supplementary Information

**Additional file 1: Table S1**. Phenotyping summary metrics of the 'O×N' population in three evaluation seasons.

**Additional file 2: Table S2**. Phenotypic characterization of six selected contrasting SSC individuals from the 'OxN' peach population in three evaluation seasons.

**Additional file 3: Table S3**. Sequencing summary metrics of selected contrasting individuals for soluble solids content phenotype.

**Additional file 4: Table S4**. Regulatory candidate genes differentially expressed between high and low SSC samples co-located with the QTL for SSC in the 'OxN' populaton.

## Data Availability

The datasets generated and analyzed during the current study are available in the National Center for Biotechnology Information (NCBI) repository, PRJNA1088620 http://www.ncbi.nlm.nih.gov/bioproject/1088620.
